# Autopsy rates in the Netherlands: 35 years of decline

**DOI:** 10.1371/journal.pone.0178200

**Published:** 2017-06-15

**Authors:** Britt M. Blokker, Annick C. Weustink, M. G. Myriam Hunink, J. Wolter Oosterhuis

**Affiliations:** 1 Department of Pathology, Erasmus University Medical Centre, Rotterdam, the Netherlands; 2 Department of Radiology and Nuclear Medicine, Erasmus University Medical Centre, Rotterdam, the Netherlands; 3 Department of Clinical Epidemiology, Erasmus University Medical Centre, Rotterdam, the Netherlands; 4 Centre for Health Decision Science, Harvard T.H. Chan School of Public Health, Harvard University, Boston, Massachusetts, United States of America; BC Children's Hospital, CANADA

## Abstract

**Objective:**

Although the autopsy still is a valuable tool in health statistics, health care quality control, medical education, and biomedical research, autopsy rates have been declining worldwide. The aim of this study was to examine trends of overall, clinical and forensic autopsy rates among adults in the Netherlands over the last four decades, and trends per sex, age (groups), and hospital type.

**Methods:**

We performed a retrospective study covering 35 years of Dutch national death counts (1977–2011), the number of in-hospital deceased patients, the number of deaths due to external causes, and the proportion of autopsies performed in these populations. The effects of sex, age and hospital category were analysed by linear and logistic regression and differences were evaluated by chi-square tests.

**Results:**

Overall autopsy rates declined by 0.3% per calendar year, clinical autopsy rates by 0.7% per calendar year (from 31.4% to 7.7%), and forensic autopsy rates did not decline. Per calendar year the fraction of in-hospital deceased patients decreased by 0.2%. Autopsy rates were highest among men and younger patients; clinical autopsy rates were highest for patients dying in academic hospitals.

**Conclusions:**

In the Netherlands clinical autopsy rates have rapidly declined while at the same time the fraction of in-hospital deaths decreased, both contributing to the overall reduced absolute number of autopsies performed. It is important to improve awareness among both clinicians and general practitioners of the significance of the clinical autopsy.

## Introduction

### Background

The relevance of the clinical autopsy is well recognized; it provides bereaved relatives with information on the cause of death and clinicians with feedback on diagnosis and treatment, thus making it an important instrument for health care quality control [1, 2]. In spite of the advanced diagnostic technologies used in modern medicine, there are still discrepancies found between clinical diagnoses and post-mortem findings [3–5] with a significant rate of class-I-discrepancies (major diagnoses) [6]. By identifying these, the autopsy improves the accuracy of both death certificates [7] and epidemiologic databases [8]. Moreover, it contributes to medical knowledge [9] provides for evidence-based research, and is a resource for biomedical research, e.g. by procurement of normal and pathological tissues [10]. Despite these benefits, clinical autopsy rates have rapidly declined worldwide in the past decades, and alternative less invasive post-mortem methods are currently being developed to improve or replace the conventional autopsy [11].

Several studies have shown local or national trends of autopsy rates [1, 12–17]. Few studies have reported on Dutch autopsy rates [2, 18] and only one study evaluated potential factors that might have influenced autopsy rates, based on a small population in the early sixties [19].

### Purpose

In this analysis of national statistics we describe the 35-year trends in the Netherlands of adult deaths, both in-hospital deceased patients and deaths due to external cause, and the clinical and forensic autopsy rates over the same period. We analysed the effects of age, sex and hospital type on the autopsy rates.

## Materials and methods

### Data collection

For each year in the period of 1977 to 2011, 35 years in all, we obtained the total number of registered adult deaths, the number of in-hospital deceased adult patients, the number of deaths due to external cause, the number of clinical and forensic autopsies performed, and if available, information on age, sex and hospital category. These variables were derived from logbooks of the Netherlands Forensic Institute (NFI), and databases provided by Statistics Netherlands (SN, a.k.a. CBS Statline) and Dutch Hospital Data (DHD) in cooperation with Kiwa Carity’s data services. The latter is a service organisation that aims to improve Dutch health care in various ways.

We analysed the SN databases for all registered adult deaths in the Netherlands per year, and we collected tables presenting the total number of deaths and the number of deaths due to external causes (S1 Table).

Kiwa Carity provided an anonymized set of aggregated data (S1 and S2 Tables), including all cases of adult patients (≥18 years) deceased in Dutch hospitals, their age and gender, the type of hospital they died in (academic or non-academic), and whether or not an autopsy was performed. Compulsory forensic autopsies in the case of suspected unnatural death, as is the policy in the Netherlands, were excluded. Using the program Matlab, we created files by year, consisting of one line per individual case. To ensure the privacy of individuals, the data were used according to the required protocol for data provided by DHD. Further ethical approval was not required for this part.

The information on performed forensic autopsies was collected from the logbooks kept by the forensic pathologists of NFI. For each case only gender and age were extracted and registered in an anonymized file. NFI has granted us ethical approval to use this file to create an overview of these forensic cases.

The emphasis of our analyses was on clinical autopsy rates. In the Netherlands, there are no extramural facilities for non-forensic autopsies. Therefore, if a person dies outside a hospital from a supposed natural cause of death, and next-of-kin ask for a post-mortem examination, the autopsy will be performed by clinical pathologists in the nearest hospital. Because this situation rarely occurs, we expect that only few cases of performed autopsies have been missed.

### Data analysis

Excel and SPSS were used for data analysis. We calculated means, differences, ratios and percentages. Overall numbers were plotted with the exact autopsy percentages, and, to filter random noise within the subgroups and make trends visible, 4-year moving average plots were constructed. Linear regression was performed to show trends over time, logistic regression to analyse the effect of possible explanatory variables (year, sex, age and hospital type), and the chi-square test to analyse differences between academic and non-academic hospitals. To identify multiple trends within the collected 35 years, we stratified the years into three time periods of 12, 12 and 11 years (1977–1988, 1989–2000 and 2001–2011). For other analyses we created age subgroups (e.g. ±20-year groups).

## Results

### General overview

**Fig 1:**
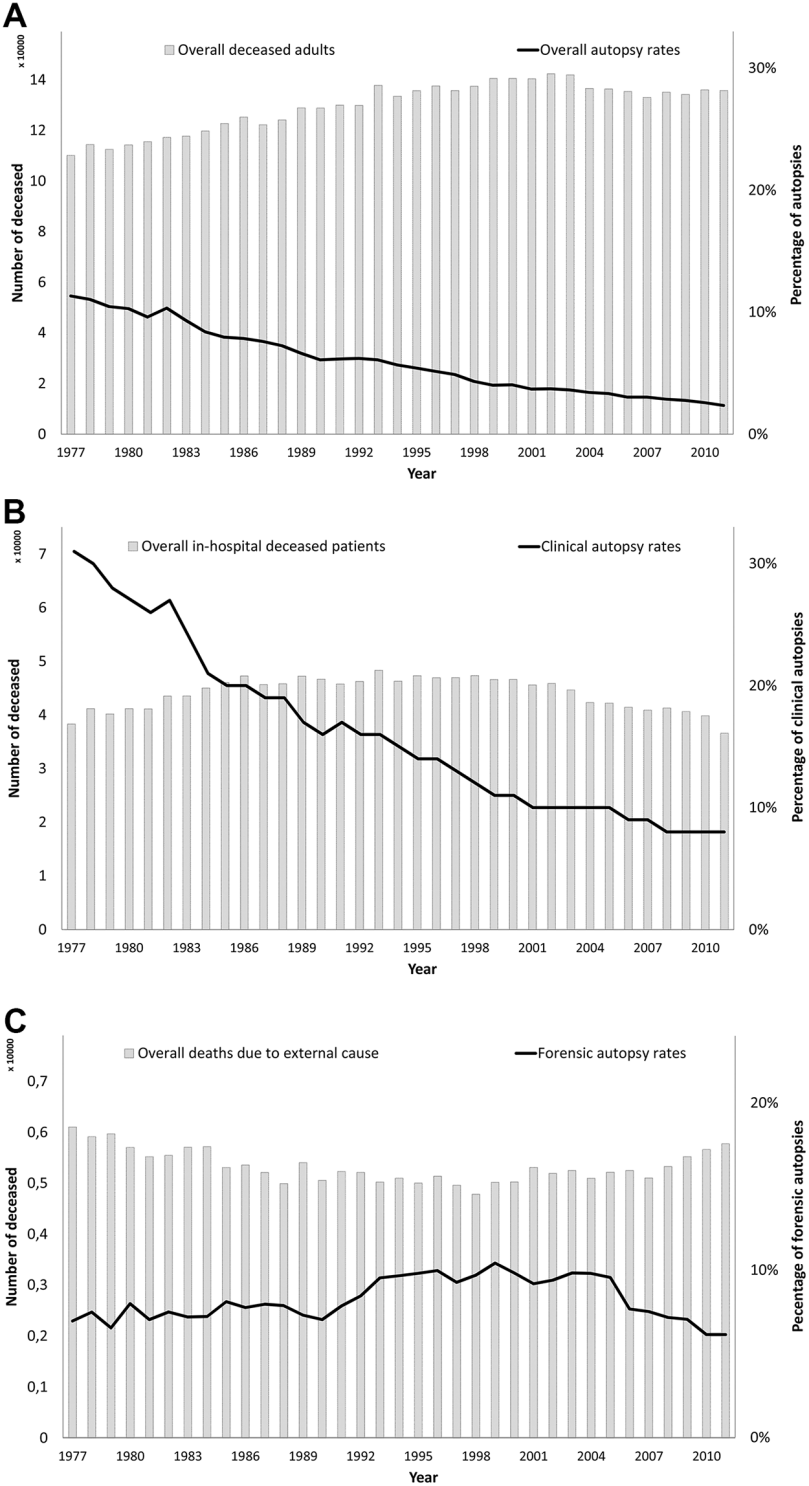
Deaths and autopsy rates per year: Overall in the Netherlands (A), clinical (B) and forensic (C).

From 1977 to 2011, 4,539,619 adults died in the Netherlands (mean: 129,703 per year, 95%CI: 110,093;142,355). The overall death counts have steadily increased with a mean of 805 per calendar year (95%CI: 640;971) and a total increase of 23.3%. The overall autopsy rates declined with 0.3% per year (Fig 1A). Each year approximately one third of these overall deceased adults died in hospitals (mean: 44,075 per year, 95%: 36,601;48,341). The percentage of in-hospital deceased shows an overall decline of 0.2% per calendar year (95%CI: -0.003;-0.002).

On 249,178 of the in-hospital deceased patients autopsies were performed (mean: 7119 per year, 95%CI: 2,820;12,209). In 35 years, the absolute number of performed clinical autopsies decreased with an average of 4% per year, to less than a quarter of the former number; each year, 282 fewer autopsies were performed (95%CI: -295;-268). Per additional calendar year, the odds of performing an autopsy on an in-hospital deceased adult patient were reduced by 5% (95%CI: 0.950;0.950). The clinical autopsy rate decreased with a mean of 0.7% per calendar year, from 31.4% in 1977 to 7.7% in 2011 (Fig 1B). When divided into the three time periods, we observed the steepest decline in the earliest period (1977–1988, Table 1).

**Table 1:** Linear regression analyses of autopsy rates, per time period per variable.

General autopsy rates		Overall	Clinical	Forensic
		Regression coefficient	Regression coefficient	Regression coefficient
		(95% CI)	(95% CI)	(95% CI)
**Time period**	**1977–1988**	-0.004	-0.012	0.001
		(-0.004; -0.003)	(-0.012; -0.012)	(0.000; 0.002)
	**1989–2000**	-0.002	-0.006	0.003
		(-0.003; -0.002)	(-0.006; -0.006)	(0.001; 0.004)
	**2001–2011**	-0.001	-0.003	-0.004
		(-0.002; -0;001)	(-0.003; -0.003)	(-0.006; -0.003)
	**1977–2011**	-0.003	-0.007	0.000*
		(-0.003; -0.002)	(-0.007; -0.007)	(0.000; 0.001)

* not significant

Only a small number of deaths each year was due to external causes (mean: 5335, 95%CI: 4,783;6,104), over the years this number decreased with 13 per year (95%CI: -23;-3). Forensic autopsy was performed in 8.5% (95%CI: 6.4–10.6, Fig 1C). The trend of forensic autopsy rates is not significant, but when divided into the three time periods we observed an increase followed by a decrease (Table 1).

### Sex of the deceased

The mean increase of overall deaths per calendar year was 705 among women (95%CI: 600;811) and 100 among men (95%CI: 37;163). Regardless of this trend, the majority of in-hospital deceased patients (54.7%, 95%CI: 52.7–57.0) and deaths due to external causes (57.9%, 95%CI: 55.0;60.0) was always male.

**Fig 2:**
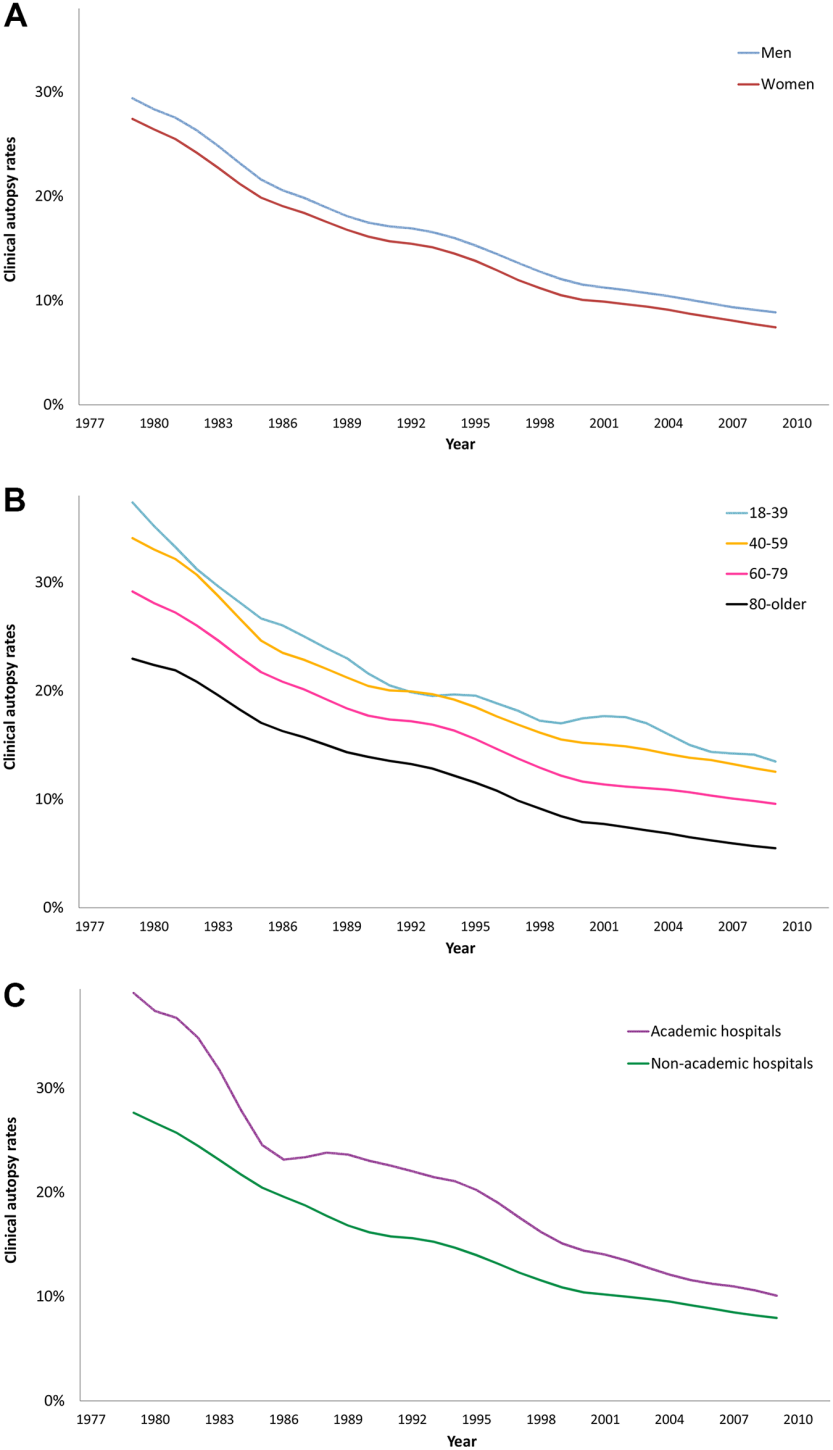
4-year moving averages of clinical autopsy rates per sex (A), age group (B) and hospital category (C).

Each year the majority of the clinical autopsies were performed on men. The number of performed clinical autopsies decreased with a mean of 167 per year (95%CI: -176;-157) among men and 115 (95%CI: -120;-111) among women. If an in-hospital deceased patient was male, the odds of performing an autopsy were higher by a factor of 15.4% (95%CI: 1.144;1.164). The difference between men and women was also present with respect to autopsy rates, both clinical and forensic. Clinical autopsy rates declined with 0.7% per year among men and 0.6% per year among women (Fig 2A). When divided into the three time periods the decline was similar between the sexes (Table 2). The forensic autopsy rates, on the other hand, showed no trend among women; only the 35-year trend among men showed a small but significant increase (0.001, 95%CI: 0.000–0.001).

**Table 2:** Linear regression analyses of clinical autopsy rates, per time period per variable.

Clinical autopsy rates		**Male**	**Female**	**Academic**	**Non-academic**
		Regression coefficient	Regression coefficient	Regression coefficient	Regression coefficient
		(95% CI)	(95% CI)	(95% CI)	(95% CI)
**Time period**	**1977–1988**	-0.012	-0.012	-0.022	-0.011
		(-0.013; -0.012)	(-0.012; -0.011)	(-0.023; -0.020)	(-0.012; -0.011)
	**1989–2000**	-0.006	-0.006	-0.009	-0.006
		(-0.006; -0.006)	(-0.007; -0.006)	(-0.010; -0.008)	(-0.006; -0.005)
	**2001–2011**	-0.003	-0.003	-0.005	-0.003
		(-0.003; -0.003)	(-0.003; -0.003)	(-0.006; -0.004)	(-0.003; -0.003)
	**1977–2011**	-0.007	-0.006	-0.009	-0.006
		(-0.007; -0.007)	(-0.007; -0.006)	(-0.009; -0.009)	(-0.007; -0.006)
		**18–39 years**	**40–59 years**	**60–79 years**	**80 years -older**
		Regression coefficient	Regression coefficient	Regression coefficient	Regression coefficient
		(95% CI)	(95% CI)	(95% CI)	(95% CI)
**Time period**	**1977–1988**	-0.016	-0.014	-0.012	-0.009
		(-0.019; -0.014)	(-0.015; -0.013)	(-0.012; -0.011)	(-0.010; -0.009)
	**1989–2000**	-0.005	-0.005	-0.006	-0.006
		(-0.007; -0.003)	(-0.006; -0.005)	(-0.006; -0.006)	(-0.006; -0.006)
	**2001–2011**	-0.007	-0.003	-0.002	-0.003
		(-0.010; -0.005)	(-0.004; -0.002)	(-0.003; -0.002)	(-0.003; -0.002)
	**1977–2011**	-0.008	-0.007	-0.007	-0.006
		(-0.008; -0.007)	(-0.007; -0.007)	(-0.007; -0.007)	(-0.006; -0.006)

### Age of the deceased

At least a quarter of young adults died in a hospital, this fraction of in-hospital deceased increases up to the age group of 69 years olds (44.5%) and then declines to less than 10%. A total of 249,178 clinical autopsies were performed, most at the age of 76. Until that age there is a mean increase of 152 autopsies per year of age (95%CI: 134;169), after that age the number of autopsies decreases by 467 per year of age (95%CI: -508;-426). Also, the autopsy rates were higher among patients who died at a younger age, with the highest peak at the age of 35. Until that age the autopsy rates increased with 0.2% per age year (95%CI: 0.001;0.003), and from the age 36 onwards the autopsy rates declined with 0.3% per age year (95%CI: -0.003;-0.003).

All four age groups showed a decline in performed autopsies. In absolute numbers most clinical autopsies were performed in the age group of 60 to 79. Autopsy rates, on the other hand, were highest in the younger age groups for both clinical autopsies (Fig 2B) and forensic autopsies. Compared to the age group of 80 years and older, the odds of a clinical autopsy being performed were 2.276 (95%CI: 2.218;2.335) among the 18–39 year age group, 1.986 (95%CI: 1.959;2.014) among the 40–59 year age group, and 1.598 (95%CI: 1.582;1.614) among the 60–79 year age group. Each calendar year the clinical autopsy rates declined within the range of 0.8% (youngest group) and 0.6% (oldest group), see Table 2.

### Hospital type

A minority of the in-hospital deceased patients died in an academic hospital, but he autopsy rates were always higher in academic hospitals than in non-academic hospitals (Fig 2C and Table 3). Over the years, academic autopsy rates declined more than non-academic autopsy rates, even when divided in the three time periods (Table 2). Compared to non-academic hospitals, the odds of an autopsy being performed in an academic hospital were 1.374 (95%CI 1.356,1.392).

**Table 3:** In-hospital deceased patients, performed autopsies and clinical autopsy rates per hospital category per time period.

		Academic			Non-academic		
		Deceased	Autopsies	Rate	Deceased	Autopsies	Rate
**Time period**	1977–1988*	46357	14524	31.33%	472431	111184	23.53%
	1989–2000*	57816	11349	19.63%	504567	69790	13.83%
	2001–2011*	52474	6001	11.44%	408984	36330	8.88%
	1977–2011*	156647	31874	20.35%	1385982	217304	15.68%

* Difference per time period P<0.001 in Chi Square-test

## Discussion

### Main findings

From 1977 to 2011 overall deaths increased, especially those among women and the age group of 80 years and older. The fraction of in-hospital deceased patients declined and there was a small decline of deaths due to external causes. Each year the majority of both the in-hospital deceased patients and deaths due to external causes were male. Also, more autopsies were performed on men. Both clinical and forensic autopsy rates were higher among men, and among patients who died at a young age (18 to 59 years).

Over the 35-year time period there was a decline of performed clinical autopsies, and a decline in clinical autopsy rates for both sexes, all age groups, and for both hospital categories. Academic hospitals performed fewer autopsies, but had higher autopsy rates than non-academic hospitals.

### Strengths and limitations of this study

We present primary results on 35 years of Dutch population-based data containing more than 4.5 million people overall, including over 1.5 million in-hospital deceased patients, and over 180 thousand deaths due to external causes. We assume that there are no missing cases, apart from those that were not officially registered with death certificates.

Of 264,450 of these cases we know that an autopsy was performed. However, it is unknown how many autopsies were performed on out-of-hospital deceased adults with a supposed natural cause of death. We assume that the number is negligible, based on publications in Dutch medical journals concerning the difference in autopsy rates between intramural and extramural diseased cases [20]. Also, from our experience we know that general practitioners or geriatricians rarely send in out-of-hospital deceased for clinical autopsy. To support this, we retrieved the numbers of autopsies performed in our own university medical centre from 2010 to 2015. We found that only 6.7% of all adult autopsies were performed on extramural cases, which correlates with the 6% reported in 1986 [21]. Overall, the autopsy rates among all extramural deaths in the Netherlands are reported to be less than 1% [21, 22].

### Comparison with the literature

According to the SN death counts in the Netherlands increased from 110,000 in 1977 to 136,000 in 2011, which could be explained by the overall population growth. There was also a relative increase from 7.9 per 1000 in 1977 to 8.1 per 1000 in 2011, which is possibly due to the substantially increased number of deceased women in the age group of 80 years and older. For years, the life expectancy at birth has been lower for Dutch men than for Dutch women, which must have led to an excess of women within the Dutch population. These women have eventually reached an older age, and passed away.

In 2003, the Dutch government eased budgetary constraints in the health care system, leading to increased health care delivery, including more active and life-prolonging treatments for the elderly [23]. As a result the life expectancy increased, and the increase of overall deaths ended.

A possible explanation for the overall decline of in-hospital deaths could be the shortening of in-hospital stays, that was initially due to budgetary constraints of the Dutch government [23] and is now continued by altered health care policy for the terminal phase of life. Ploemacher et al. suggested that patients are currently more often discharged from hospitals to receive palliative care from external facilities [24] and as a result more patients die at home or in nursing homes. The decline of in-hospital deceased could further be explained by an increase of deaths due to cancer, especially within the age groups of 60 years and older. According to Van der Wal et al. a substantial number of cancer patients (48%) died at home [25]. A factor possibly related to the excess of in-hospital deceased men (and performed autopsies), is that men more often have health problems that correlate with higher mortality rates, whereas women have health problems with a higher disease burden [26].

As a direct result of decreasing in-hospital deaths, fewer autopsies were performed in the Netherlands. Also the autopsy rates declined, just as observed in other countries [1, 13, 27], especially with increasing age of the deceased [13, 27]. Among the age group of 60 to 79 years fewer autopsies were performed each year, which might be correlated with the increasing number of deaths due to cancer that is observed in that same age group. If a patient dies of cancer, the cause of death seems obvious to next-of-kin [28] and an autopsy superfluous.

At the same time, the clinician might be less eager to ask for an autopsy [29] especially if end-of-life decisions were made and euthanasia was performed. The requirements for requesting euthanasia in the Netherlands are extensive, for instance, it has to be shown that the disease is intolerable and that treatment options are lacking [25]. To support this contention, the clinician must have documented all diagnoses and therapy options carefully, and may feel that the need for autopsy is less urgent. Hence, the decline in autopsy rates is multifactorial and cannot be explained only by fewer consents from next-of-kin. This conclusion is supported by Gaensbacher et al., who observed declining autopsy rates in Austria, where no consents are needed for clinical autopsies [13].

Autopsy practices differ per country, for example policies on financing autopsy, the rate of forensic autopsies, sites where autopsies are performed, and the necessity of obtaining consent from next-of-kin.

Financing of the clinical autopsy is complicated [30]. Data on the exact costs per autopsy are not available; cost estimates per autopsy vary according to the number of autopsies being performed [31] and the extensiveness of the procedure [32]. At the same time gained benefits per autopsy are difficult to quantify, and, as a consequence, cost-benefits of autopsy cannot easily be determined. Due to competing business activities and scarce health care resources, autopsy financing appears not to be a priority of today’s hospitals [33]. It is often not clear from which departmental or institutional budget the autopsy costs are, or should be, derived. The lack of a firm financial basis for autopsy services has very likely contributed to declining autopsy rates [12]. In Dutch hospitals, however, the costs for autopsy are paid off the general hospital budget. There are neither financial nor capacity constraints for clinicians or next of kin to have an autopsy performed, therefore, financial and capacity issues cannot explain the decline of the autopsy rate in the Netherlands.

There are also different policies for financing the medicolegal/ forensic autopsy. For example, in Denmark forensic autopsies are paid from the police budget and thus compete with other cost [32], whereas in Finland the forensic autopsies are all payed for by the government. Even in recent years, the overall Finnish autopsy rates have been around 30%, which is explained by increasing medicolegal autopsy rates at the time when clinical autopsy rates started to decrease.

In the Netherlands, non-forensic autopsy cases with supposed natural death are carried out in general hospitals, whereas in the investigated period forensic cases were performed at NFI. In some countries, however, forensic autopsy may also be performed on cases that are not of interest to the police, such as deceased whose cause of death is classified as natural, but remains unclear [32].

In many countries consent from next-of-kin is compulsory for a non-forensic autopsy, however in some countries, autopsy may be performed without consent (if there is a clear medical or scientific interest [13]). In some other countries, next-of-kin may object to autopsy even though consent for autopsy is not required; so-called opt out-system. In few countries autopsy has even been mandatory [34].

**Fig 3:**
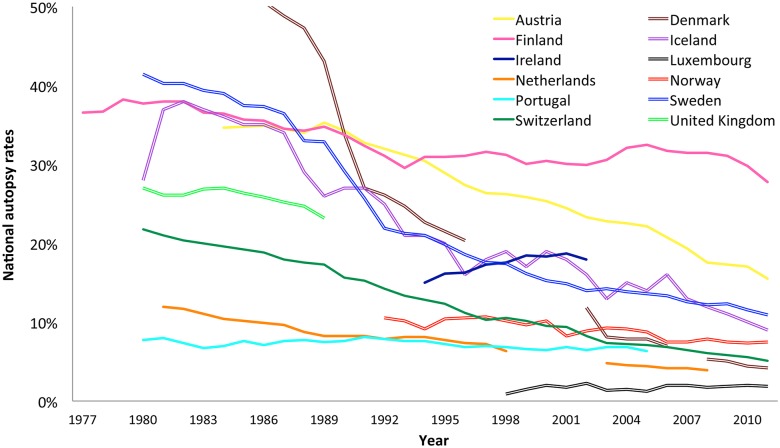
National autopsy rates of Western European countries according to the World Health Organization (European Health Information Gateway).

Despite these and other policy differences, the general trend is declining autopsy rates. To illustrate this, we plotted national autopsy rates of Western European countries during the investigated time period, using overall autopsy rates collected from the WHO European Health Information Gateway, including deceased under 18 years of age (Fig 3, S3 Table).

Since we included adult cases only, our clinical autopsy rates are somewhat different from those reported in the literature. Fetuses and neonates are usually more often autopsied than adults [35].

Autopsy rates were consistently higher for men than women. This phenomenon is also seen in other studies [14, 36] and one could wonder why. Is it because men are usually younger than women, when they die? Do we try harder to explain the cause of death in men than that in women? Are bereaved wives more willing to give consent, than bereaved husbands?

That autopsy rates were higher in academic hospitals than in non-academic hospitals was expected [17, 31]. Patients in academic hospitals generally have more complex pathologies than those in non-academic hospitals. If such patients die, it is more likely that the clinicians (and next-of-kin) feel the need for post-mortem investigation. In addition, academic doctors might have a more active approach to (further) investigation, than specialists in non-academic hospitals. Also, the teaching and research responsibilities in the academic hospital are probably in favour of autopsies.

Various other explanations for the (worldwide) declining autopsy rates have been mentioned, such as religious or cultural convictions of both doctors and next-of-kin, funeral delay, fear for mutilation of the deceased’s body, absence of a defined minimum autopsy rate, cost reduction policies, pathologist’s resistance to autopsy, adverse media attention [1, 9, 37, 38] and improved pre-mortem diagnostic techniques. It is generally assumed that the decline of autopsy rates in the recent years was speeded up by the improved diagnostic value of the imaging techniques.

In our study, however, linear regression showed the largest decline of clinical autopsy rates in the first time period (1977–1988), when the two revolutionary new imaging techniques had not yet been implemented in Dutch hospitals. In the seventies ultrasound and endoscopic techniques were introduced in clinical practice, but due to restrictive governmental policies, computed tomography (CT) was introduced relatively late. Only since the late eighties all radiology departments in Dutch hospitals had a CT-scan, and at that same time magnetic resonance imaging (MRI) was introduced [39]. We hypothesize that the imaging techniques improved along with many other diagnostic techniques, and that together they may have led to the phenomenon of overconfident clinicians [40] who underestimate the relevance of clinical autopsy. This was confirmed in a recent study, which showed that the main reason for clinicians not to request an autopsy was the assumption that the cause of death was known [28].

To revive the interest of clinicians in the autopsy with its various significant applications is medicine, we may as well use these improved imaging techniques to our advantage. If, in the future, next-of-kin refuse conventional autopsy, clinicians could offer them alternatives, whereby state of the art imaging is the basis of a minimally invasive autopsy technique. Recently, the feasibility of both non-invasive and minimally invasive approaches, using CT and/ or MRI as alternatives for the autopsy, is being investigated [41, 42]. With minimally invasive autopsy techniques tissue biopsies can be obtained for histologic examination and molecular analyses [10].

Importantly, these alternatives may be more acceptable to populations that have fundamental problems with the conventional autopsy. Epidemiology might also benefit from introduction of imaging-based post-mortem investigation, because it makes a snapshot and a permanent record of the deceased that can be revisited as new questions arise.

## Conclusions

Clinical autopsy rates have been declining rapidly, probably most of all because clinicians are convinced that the autopsy will not show anything other than what is already known through pre-mortem diagnostics. This is a major concern, because autopsies to this day disclose findings that might have changed the treatment of the patient, in addition to being an important tool for quality control, education and research in medicine. Efforts should be made to revive the interest in the clinical autopsy, in particular by introducing approaches whereby state of the art imaging is integrated with a minimally invasive autopsy technique.

## Supporting information

S1 Table Overview of cases in SN and DHD databases. (XLSX)

S2 Table SPSS database of all individual cases provided by Dutch Hospital Data (DHD) in cooperation with Kiwa Carity’s data services. (SAV)

S3 Table Overall autopsy rates collected from the WHO European Health Information Gateway (https://gateway.euro.who.int/en/indicators/hfa-indicators/hfa_545-6410-autopsy-rate-for-all-deaths/). (XLSX)
